# The Vitamin A and D Exposure of Cells Affects the Intracellular Uptake of Aluminum Nanomaterials and Its Agglomeration Behavior: A Chemo-Analytic Investigation

**DOI:** 10.3390/ijms21041278

**Published:** 2020-02-14

**Authors:** Fabian L. Kriegel, Benjamin-Christoph Krause, Philipp Reichardt, Ajay Vikram Singh, Jutta Tentschert, Peter Laux, Harald Jungnickel, Andreas Luch

**Affiliations:** German Federal Institute for Risk Assessment, Department of Chemical & Product Safety, Max-Dohrn-Straße 8-10, 10589 Berlin, Germany; Benjamin-Christoph.Krause@bfr.bund.de (B.-C.K.); Philipp.Reichardt@bfr.bund.de (P.R.); Ajay-Vikram.Singh@bfr.bund.de (A.V.S.); Jutta.Tentschert@bfr.bund.de (J.T.); peter.laux@bfr.bund.de (P.L.); Harald.Jungnickel@bfr.bund.de (H.J.); Andreas.Luch@bfr.bund.de (A.L.)

**Keywords:** nanoparticle uptake, ICP-MS, ToF-SIMS, aluminum, vitamin, metabolomics

## Abstract

Aluminum (Al) is extensively used for the production of different consumer products, agents, as well as pharmaceuticals. Studies that demonstrate neurotoxicity and a possible link to Alzheimer’s disease trigger concern about potential health risks due to high Al intake. Al in cosmetic products raises the question whether a possible interaction between Al and retinol (vitamin A) and cholecalciferol (vitamin D3) metabolism might exist. Understanding the uptake mechanisms of ionic or elemental Al and Al nanomaterials (Al NMs) in combination with bioactive substances are important for the assessment of possible health risk associated. Therefore, we studied the uptake and distribution of Al oxide (Al_2_O_3_) and metallic Al^0^ NMs in the human keratinocyte cell line HaCaT. Possible alterations of the metabolic pattern upon application of the two Al species together with vitamin A or D3 were investigated. Time-of-flight secondary ion mass spectrometry (ToF-SIMS) imaging and inductively coupled plasma mass spectrometry (ICP-MS) were applied to quantify the cellular uptake of Al NMs.

## 1. Introduction

Aluminum is one of the most abundant metals that is used in a wide range of industrial manufacturing processes. It is also present in numerous consumer products such as cosmetics or food contact materials. Studies on Al toxicity revealed a potential risk for neuronal toxicity in humans following chronic Al exposure [1,2] and a possible relation of enhanced Al intake to the development of Alzheimer’s disease [3].

The uptake of Al may occur via different routes of exposure. Al NMs (nanomaterials) may cross the barriers of the body because of their small size and thus can significantly increase the overall Al burden [4]. Humans are most likely exposed to Al through cosmetic products due to skin contacts or via food additives [3]. To lower the intake of Al, the first legal actions were taken by the EU regulation No. 380/2012 amending Annex II to Regulation (EC) No. 1333/2008, which became applicable at 1st of August 2014 [5]. Further to this, the use of Al-containing food additives is restricted by the recommendation of the European Food Safety Authority (EFSA) to lower the tolerable weekly intake (TWI) of Al to ≤1 mg/kg body weight. However, it was suggested that this TWI might be significantly exceeded, especially in children [6].

To avoid such a high intake, the conditions simulating use and tolerable quantities for food additives containing Al were adapted in the EFSA regulation. The body’s uptake of Al is influenced by several processes. For example, vitamin D3 not only enhances the uptake of essential inorganic elements but also of non-essential and toxic elements such as lead or Al [7]. Vitamin D3 also interferes with retinol metabolism and its uptake in human epidermal keratinocytes [8]. The studies related to the interaction between vitamin D3/A and Al may comprise, among other biological endpoints, Al-mediated toxicity, its distribution pattern at the cellular level, as well as its capability to modulate metabolic patterns of skin cells. The complexation behavior of Al is highly sophisticated and certainly adds to the challenges and elucidation of uptake mechanisms. Furthermore, the ubiquitous occurrence of Al resulting in a high background level hampers the application of sensitive analytical methods. The relatively low density of the light metal, its low mass and the complexity of biological matrices complicate the use of widespread analytical techniques like Raman spectroscopy or transmission electron microscopy (TEM). ToF-SIMS analysis [9] has been previously applied for the detection of cerium dioxide particle clusters in rat lung tissue [10] and for the characterization of Al particles in artificial saliva [11]. In this study, we tried to overcome the mentioned analytical limitations by using ToF-SIMS to explore uptake and distribution patterns of Al and Al_2_O_3_ NMs in the human keratinocyte cell line HaCaT. Furthermore, metabolic profile changes of the cell membrane constituents were investigated. Aluminum chloride (AlCl_3_·6H_2_O) was used as soluble ionic control. All three Al species were tested with regard to uptake, cellular distribution patterns, and possible cell membrane alterations upon uptake. In addition to single applications, combinations with vitamin A and D3 were tested as well. ICP-MS measurements were utilized to determine the uptake efficacy of each Al species in the different scenarios.

## 2. Results and Discussion

### 2.1. Characterization of Al and Al_2_O_3_ NMs

Both, Al^0^ and Al_2_O_3_ NMs have been extensivly characterized by our group [12] (see Table 1). The core particle diameter was determined by means of TEM to be between 2–50 nm for the rather spherical Al^0^ NMs. Single particle (SP) ICP-MS showed a primary particle size range of 50–80 nm for both Al NMs. The results of the small angle x-ray scattering (SAXS) measurments confirmed the findings of TEM and SP-ICP-MS. 

**Figure 1:**
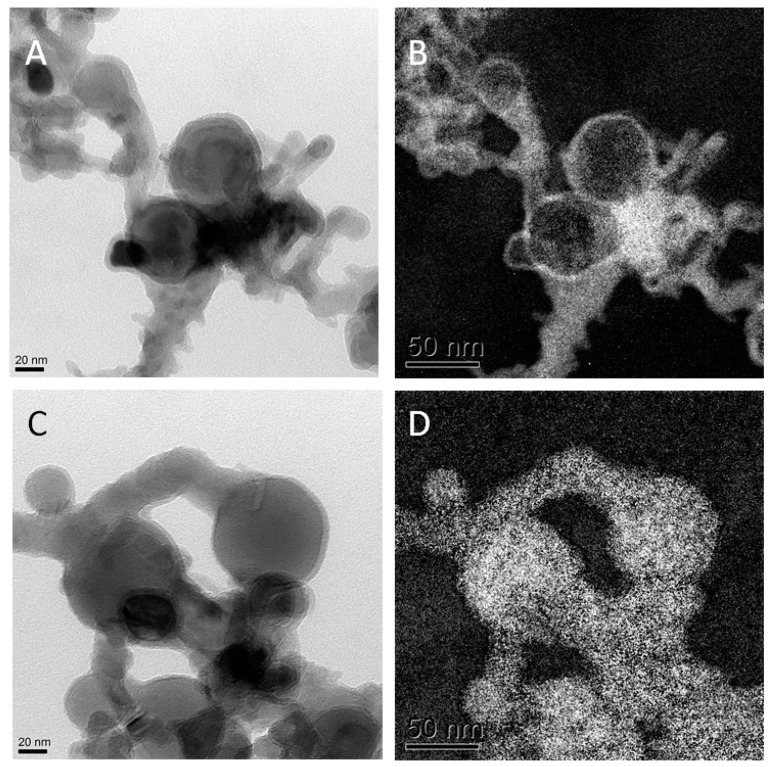
Transmission electron microscopy (TEM) results: (**A**) TEM pictures of Al^0^ NMs; (**B**) oxygen mapping of left TEM picture; (**C**) TEM picture of Al^0^ NMs; (**D**) aluminum mapping of image in (**C**).

The more rod shaped Al_2_O_3_ NMs display a width of 10 nm and a length between 20–50 nm as determined by TEM. Dynamic light scattering (DLS) determined the hydrodynamic diameter in dispersion of 250 nm for Al^0^ NMs, while Al_2_O_3_ NMs showed a smaller diameter of 180 nm. Zeta Potential measurements of Al^0^ and Al_2_O_3_ NM showed comparable results with −17.2 mV and −17.3 mV in DMEM. X-ray diffraction (XRD) as well as TEM in electron energy loss spectroscopy mode (EELS-TEM) demonstrated the difference of the Al^0^ and Al_2_O_3_ NMs composition and especially their surface. While Al^0^ NMs had a core-shell structure with Al core and a 2–5 nm oxygen shell (see Figure 1), the Al_2_O_3_ NMs were homogeneously oxidized.

### 2.2. Cellular Uptake

**Figure 2:**
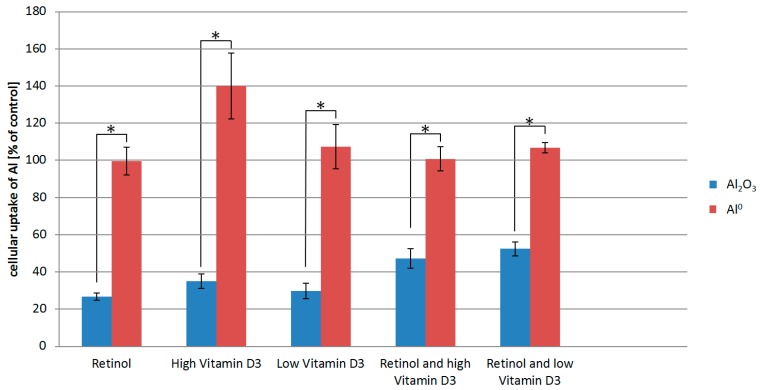
Inductively coupled plasma mass spectrometry (ICP-MS) measurements of Al content of HaCaT cells exposed to either Al_2_O_3_ or Al^0^ NMs as well as retinol and/vitamin D3. The cellular uptake is normalized to the Al uptake of cells exposed to Al^0^ NMs or Al_2_O_3_ NMs only. * *p* < 0.05.

To investigate the possible uptake of Al NMs and Al_2_O_3_ NMs, we exposed HaCaT cells to 100 µg/mL Al NMs for 24 h in the presence or absence of 1 µmol/L retinol, 5.12 µmol/L of vitamin D3 (high vitamin D3), or a lower vitamin D3 concentration of 80 nmol/L (low vitamin D3). Subsequently we analyzed the exposed cells using ICP-MS to quantify the NM uptake. Quantitative results are shown in Figure 2. Untreated cells had Al levels comparable to ICP-MS blank samples.

For the interpretation of the results, it has to be considered that the data shown are normalized to the Al^0^ uptake by HaCaT cells exposed to Al^0^ only (without co-exposure to vitamins). The ICP-MS results show that retinol and vitamin D3 appear to have no detectable effect on the uptake of the Al^0^ NM. In contrast, treatment of HaCaT cells with retinol or vitamin D3 significantly lowered the uptake of the Al_2_O_3_ NM (Figure 2). 

The different uptake behavior of the two kinds of Al NMs after treatment of the HaCaT cells with vitamins is likely due to differences in their physicochemical properties. It is known that the complexation behavior and thus the agglomeration rate of the used Al NMs differ strongly [12]. Al_2_O_3_ is characterized as a rather insoluble and rod shaped oxidized NM, whereas Al^0^ NMs are partially soluble and quasi-spherical. Differences in the particle physicochemical properties (e.g., surface area, solubility, etc.) may lead to differences in the uptake mechanism preferred by the cells.

These findings presented in this study are in good accordance with the results of an uptake study of polystyrene NMs on Caco-2 cells [13]. Furthermore, the composition of the NMs might also influence their uptake and distribution [14]. The core-shell structure of the Al^0^ NM (see Table 1) contains approximately 85% Al. In contrast, Al_2_O_3_ is fully oxidized and shows a homogenous distribution of Al as well as oxygen on its surface. Our group was able to demonstrate that the protein corona that is formed during the contact of NMs with cell culture media is less complex for Al^0^ NM compared to the protein corona of Al_2_O_3_ NM [15]. The surface properties of NMs facilitate interactions with the surrounding environment, which also affects the bioavailability and the interactions of the NM with the cell.

**Figure 3:**
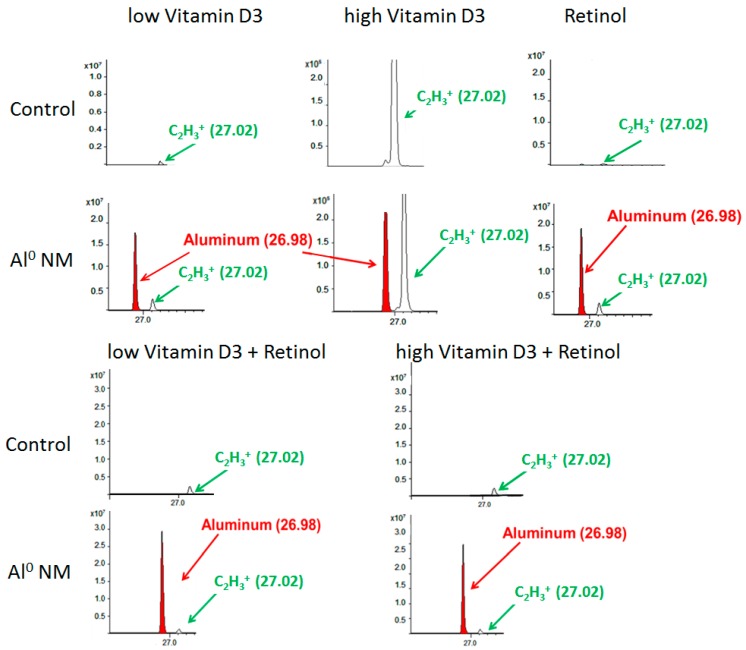
Time-of-flight secondary ion mass spectrometry (ToF-SIMS) mass spectrum (positive mode), showing the Al peak in red color (at *m*/*e* 26.98 u) and a peak in green color (at 27.02 u = C_2_H_3_^+^), resulting from organic matter in HaCaT cells. The upper line shows the spectra for control HaCaT cells, the lower line for HaCaT cells which were exposed to Al NMs (about 20 nm) for 24 h in addition to high or low vitamin D3, and retinol or their combinations. The x-axis shows the mass to charge ratio (*m*/*z*); y-axis the ion intensities.

For the assessment of changes of the metabolite patterns of the HaCaT cell membranes and the overall particle distribution, ToF-SIMS analytics was employed. The acquired ToF-SIMS mass spectra for HaCaT cells showed a strong Al peak indicating the presence of Al NMs (Figure 3). Cellular uptake can also be observed in HaCaT cell cultures co-incubated with retinol, vitamin D3, and its combinations. The Al NM uptake could also be observed after treatment with Al_2_O_3_ as well as in cultures cultivated with retinol plus vitamin D3 low or high for both NM species.

3D reconstruction of ToF-SIMS images from single HaCaT cells reveals the intracellular presence of Al^0^ and Al_2_O_3_ NMs at all exposure scenarios: NM alone, NM in combination with retinol and/or vitamin D3.

**Figure 4:**
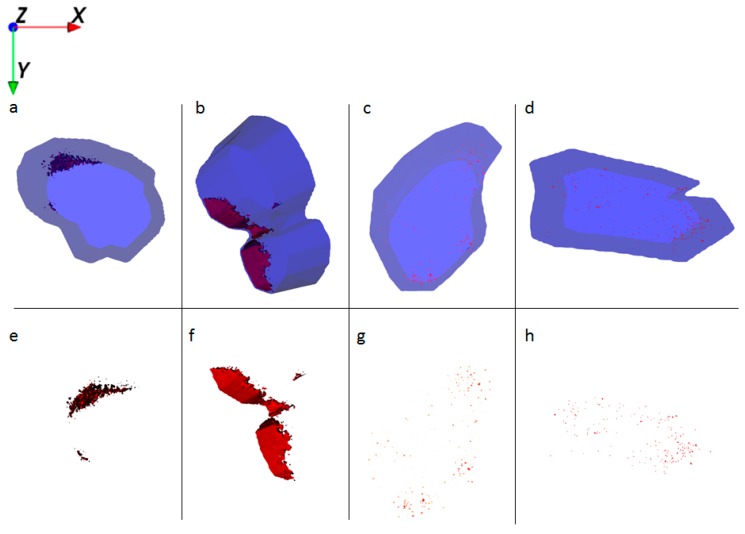
Ion reconstruction of a 3D depth profile (depth layer numbers: 50–250) of one single HaCaT cell, which was exposed to Al^0^ NMs for 24 h. The images show the top-down view of the outline of a cell of a depth profile. The translucent blue outline was reconstructed based on the C_3_H_8_N^+^ signal that originates from intracellular amino acids. (**a**) Control cells treated with Al^0^ NM only (red color); and corresponding intracellular localization of Al^0^ NM agglomerates (**e**). (**b**) Cells treated with retinol and Al^0^ NM (red color); and corresponding intracellular localization of Al NM agglomerates (**f**). (**c**) Cells treated with low vitamin D3 and Al^0^ NM (red color); and corresponding intracellular localization of Al^0^ NM agglomerates (**g**). (**d**) Cells treated with high vitamin D3 and Al^0^ NM (red color); and corresponding intracellular localization of Al^0^ NM agglomerates (**h**).

HaCaT cells treated with Al NMs only store the particles in large agglomerates (Figure 4a,e), whereas cells treated with Al NMs in combination with retinol or vitamin D3 show a different uptake and distribution behavior (compare Figure 4b,f vs. Figure 4c,g). Treatment with retinol leads to the accumulation of Al close to the cell membrane with large agglomerates (Figure 4b,f). This process might be due to the increased collagen synthesis and reduced matrix metalloproteinase expression which is known to occur because of vitamin A treatment [16]. The resulting collagen increase facilitates NM collagen interactions [17] which might hinder the NM allocation. Upon co-application of vitamin D3 the ToF-SIMS analyses revealed a different cellular deposition pattern of Al^0^ NMs when compared to that after co-exposure to retinol. Co-exposure with vitamin D3 led to an even distribution of particles throughout the entire cell and to the formation of much smaller aggregates (Figure 4c,d,g,h). In addition of being responsible for an enhanced uptake of metals (Figure 2), vitamin D3 also seems to affect particulate distribution patterns within the cell. The ToF-SIMS results are in accordance with the ICP-MS findings, where high vitamin D3 is responsible for an enhanced intracellular uptake (Figure 2).

**Figure 5:**
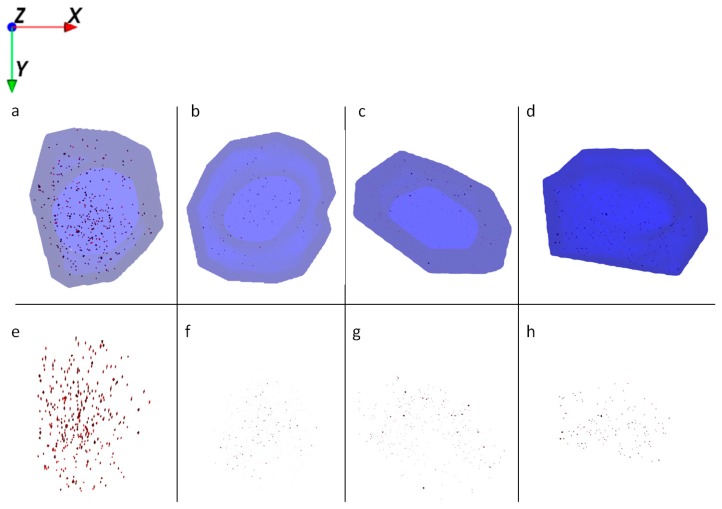
Ion reconstruction of a 3D depth profile (depth layer numbers: 50–250) of one single HaCaT cell exposed to Al_2_O_3_ NMs for 24 h. The images show the top-down view of the outline of a cell of a depth profile. The translucent blue outline was reconstructed based on the C_3_H_8_N^+^ signal that originates from intracellular amino acids. (**a**) Control cells exposed to Al_2_O_3_ NMs only (red color); and corresponding intracellular localization of Al_2_O_3_ NM agglomerates (**e**). (**b**) Cells treated with retinol and Al_2_O_3_ NMs (red); and corresponding intracellular localization of Al_2_O_3_ NM agglomerates (**f**). (**c**) Cells treated with low vitamin D3 and Al_2_O_3_ NMs (red); and corresponding intracellular localization of Al_2_O_3_ NM agglomerates (**g**). (**d**) Cells treated with high vitamin D3 and Al_2_O_3_ NMs (red); and corresponding intracellular localization of Al_2_O_3_ NM agglomerates (**h**).

The ToF-SIMS results for the exposure of HaCaT cells with Al_2_O_3_ NMs show that either treatment with retinol or vitamin D3 leads to a strong decrease in the NM uptake and to smaller agglomerate sizes when compared to cells treated with Al_2_O_3_ NMs only (Figure 2). Comparison of Al^0^ and Al_2_O_3_ NM treatments of HaCaT cells reveals a generally smaller size of NM agglomerates in the latter ones (Figure 5). Based on this it can be assumed that the number of particle agglomerates per cell is much higher for Al_2_O_3_ than for Al^0^ NMs. Therefore Al_2_O_3_ NM aggregation is being largely compromised resulting in a more even intracellular distribution of Al_2_O_3_ NMs when compared with Al^0^ NM (compare Figure 4b,f vs. Figure 5b,f).

**Figure 6:**
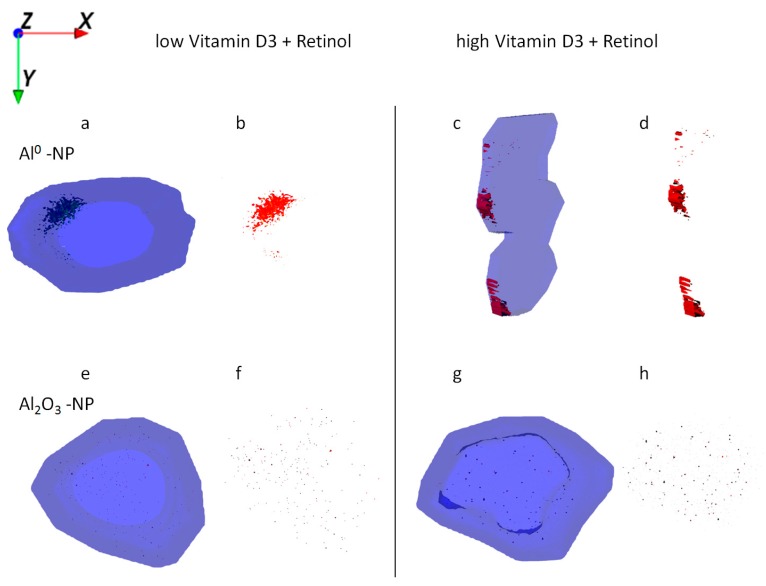
Ion reconstruction of a 3D depth profile (depth layer numbers: 50–250) of one single HaCaT cell exposed to Al NMs (upper panel **a**–**d**) or Al_2_O_3_ NMs (lower panel **e**–**h**). The 3D depth profile of the cell is depicted as translucent blue. The images show the top-down view into the outline of a cell of a depth profile. In addition to NM treatment low vitamin D3 (**a**,**b**,**e**,**f**) and high vitamin D3 (**c**,**d**,**g**,**h**) were administered together with retinol. NM agglomerates are shown in detail next to their respective cell.

When HaCaT cells were exposed to retinol in combination with either low or high levels of vitamin D3 and Al, the localization of the particles was restricted to the cell membrane region (Figure 6a,c), which corroborates above findings in Figure 4 and Figure 5. ICP-MS results show, however, a similar mass balance for all exposure experiments with Al NMs (Figure 2), indicating again a much larger number of smaller agglomerates in the case of exposure to vitamin D3.

Uptake of Al_2_O_3_ NMs alone and in combination with retinol, vitamin D3, or both vitamins revealed wide distribution and agglomeration of Al nanomaterials throughout the cytoplasm. In contrast to all co-exposure experiments, formation of larger NM clusters was observed after exposure to Al_2_O_3_ NMs alone (Figure 5a,e). ICP-MS data show a significant increase of Al_2_O_3_ NMs being present in HaCaT cells following their straight application in contrast to the co-exposure experiments (Figure 2). In addition, ToF-SIMS results show a significant reduction in the sizes of NM agglomerates after the co-exposure experiments (Figure 5 vs. Figure 6).

### 2.3. Metabolic Changes after Nanomaterial Uptake

**Figure 7:**
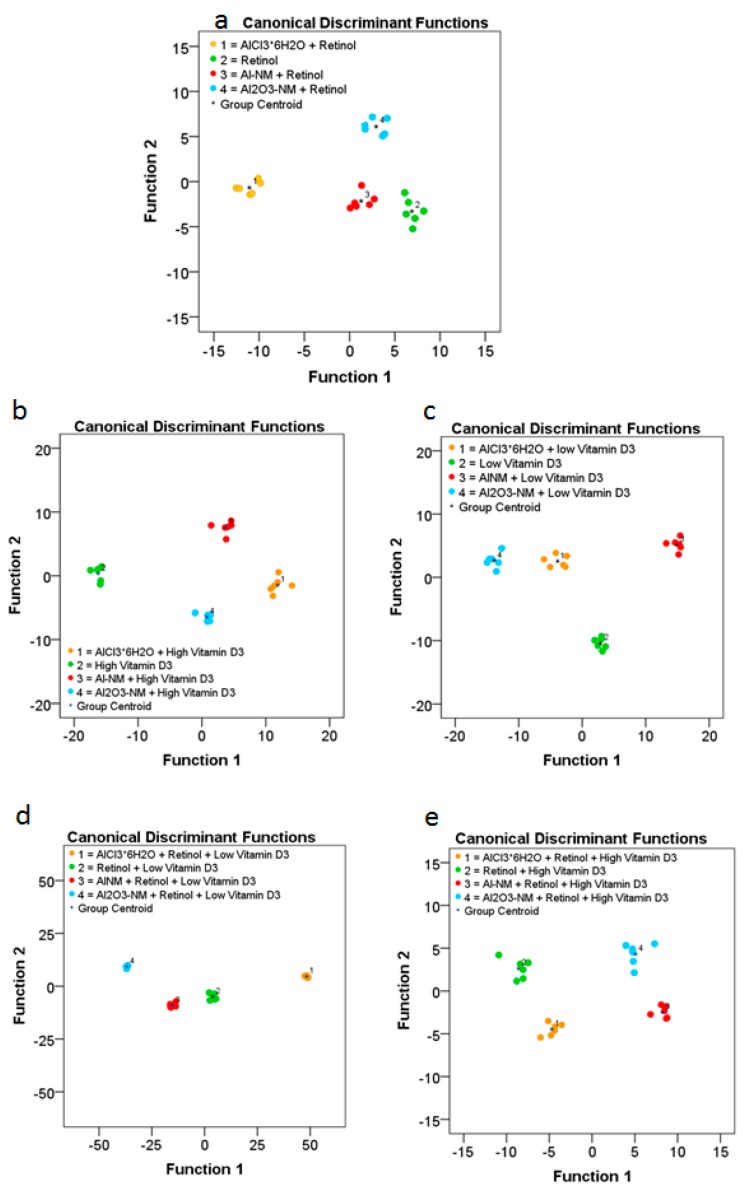
ToF-SIMS analysis of changes in the composition of the cell membranes of HaCaT cells after treatment with Al or Al_2_O_3_ NMs and ionic AlCl_3_·6H_2_O without or in combination with vitamins ((**a**) retinol; (**b**) high vitamin D3; (**c**) low vitamin D3; (**d**) retinol plus low vitamin D3; (**e**) retinol plus high vitamin D3). The diagram shows the values of the discriminant scores obtained from Fisher’s discriminant analysis of 24 single HaCaT cells for each experiment. The performance of the discriminant model was verified by applying the cross-validation procedure based on the “leave-one-out” cross-validation formalism (100%).

In addition to NM uptake and distribution, we assessed the alterations of the cell membrane constituents of HaCaT cells caused by Al and Al_2_O_3_ NM exposures. Treatments with ionic AlCl_3_*6H_2_O and unexposed HaCaT cells were used for comparison (Figure 7).

The results obtained for cells exposed to retinol, low vitamin D3, and high vitamin D3 reveal significant differences in the composition of the respective cell membranes (Figure 7). Significant differences in the cell membrane composition could also be observed in HaCaT cells co-exposed to retinol and low vitamin D3 or high vitamin D3. In order to determine the differences of lipid membrane constituents we further investigated the significant changed metabolites upon co-epxosure of Al^0^ or Al_2_O_3_ NMs with retinol or high or low vitamin D3 (see Table 2 for Al^0^ and Table 3 for Al_2_O_3_). For further details please see also the Supplementary Material (Figures S1–S6).

The further investigation of changes in the membrane composition was carried out via ToF-SIMS analysis of the major altered cell membrane lipids. Diacylglycerols (DAGs) and phosphatidic acids (PAs) were found to be increased following treatment with either Al^0^ or Al_2_O_3_ NMs in combination with vitamins similarly (see Table 2 and Table 3). DAGs were significantly increased after administration of retinol or vitamin D3 together with both Al NMs. DAGs are the product of hydrolysis of the phospholipid phosphatidylinositol 4,5-bisphosphate (PIP2) that serve as activators of the protein kinase C (PKC) pathway [18]. Alterations in the PKC signaling in HaCaT cells lead to strong morphological changes including shape of this cell type [19]. Different PKC isoforms have also different effects on the proliferation and differentiation behavior of HaCaT cells [20]. The PAs and its precursor substances lyso-phosphatidic acid were significantly increased over all treatment regimes. The PAs can be degraded to DAGs and serve as precursors of other membrane lipids [21]. Therefore, we conclude a comparable metabolic alteration of DAGs and PAs in response to Al NM uptake. Phosphatidylethanolamines (PEs) are another important group of membrane constituents. The PEs serve as substrates for phosphatidylcholine (PC) biosynthesis and are crucial for cell surface signaling [22]. Investigations on the ratio of PC to PE in mice showed decreased membrane integrity and the development of a leaky membrane when the ratio decreases [23]. In D3-treatments an increase of the level of PEs was detected because of a shift of the PCs toward PEs thereby causing a leaky membrane of the cells affected. High vitamin D3 addition revealed significantly increased levels of lyso-PC, which is a degradation product of PC and therefore indicates reduced PC levels. This process lowers the PC to PE ratio even more drastically and an enhanced uptake of Al may occur. These findings fit well with the ICP-MS results (see Figure 2). 

The significantly increased metabolites after treatment with Al_2_O_3_ and retinol or vitamin D3 are more diverse when compared to Al^0^-treated cells (see Table 3).

In addition to the above mentioned findings, another metabolite, dihydroceramides (DCs), was found to be enhanced after the treatment of HaCaT cells with Al_2_O_3_ and vitamin D3 (see Table 3). The increased levels of this compound in HaCaT cells exposed to Al_2_O_3_ NM and vitamin D3 provides another explanation for the decreased nanoparticle uptake in cells exposed to Al_2_O_3_ NM as DCs enhance the rigidity of the plasma membrane [24]. This could lead to changes in the active transport, vesicle formation, diffusion, and activation of the cell-signaling pathway, all of which are representing processes that depend on plasma membrane dynamics [25]

The changes in the membrane lipid composition described above serve as an explanation for the decreased uptake of Al_2_O_3_ NMs after vitamin A/D3 treatment. The increased levels of DCs after treatment with vitamin D3 could also derive from a protective mechanism of the cell to secure the physiological integrity of mitochondria. It has been previously shown that DCs block the permeabilization of the mitochondrial outer membrane [26].

The comparison of the changes in the constituents of the cell membrane that were introduced upon treatment with either Al^0^ or Al_2_O_3_ NMs clearly shows the necessity to distinguish between the different types of aluminum NMs. 

## 3. Materials and Methods 

### 3.1. Cell Culture and NM Exposure

Al NMs (18 nm, 99.9%) and Al_2_O_3_ NMs (20 nm, 99+%) were purchased from IoLiTec Ionic Liquids Technologies GmbH, Heilbronn, Germany. The following chemicals were purchased from Sigma-Aldrich (Sigma-Aldrich Corp., St. Louis, MO, USA): AlCl_3_·6H_2_O (hexahydrate, ≥97%), retinol (≥97.5%), cholecalciferol (certified reference material), and calcipotriol (European Pharmacopoeia Reference Standard). The chemicals were diluted to the respective concentrations (high vitamin D3 high concentration: 5.12 µmol/L; low vitamin D3 concentration: 80 nmol/L; retinol: 1 µmol/L) in DMSO (≥99.7%), obtained from Sigma-Aldrich (Sigma-Aldrich Corp., St. Louis, MO, USA). 

The human immortalized keratinocyte cell line HaCaT was cultured in Dulbecco’s modified Eagle’s medium (DMEM), 10% fetal bovine serum, and 1% antibiotics (10,000 μg/mL streptomycin and 10,000 units/mL penicillin) at 37 °C with 5% CO_2_. Cells were passaged at 70–80% confluence two times a week. 

For ToF-SIMS measurements 0.05 × 10^6^ cells were seeded on 1 cm^2^ silica wafers and left in the incubator for 24 h. Afterwards cells were treated with the respective NM with or without vitamin derivates for 24 h. The wafers were then washed using 150 mM ammonium bicarbonate solution. Samples were fast frozen and lyophilized prior to ToF-SIMS measurements.

NM dispersions were prepared following the NanoGenoTox dispersion protocol: “Final protocol for producing suitable manufactured NMs exposure media” (October, 2011). In brief, a 2.56-mg/mL stock dispersion of each NM was prepared by pre-wetting the powder with 0.5% (vol/vol) ethanol (96%) followed by addition of Millipore water containing 0.05% BSA. Dispersion occurred for 5 min and 9 s at an amplitude of 10% with a probe sonifier, either 200 W Bandelin Sonopuls HD 2200, (BANDELIN Electronic GmbH & Co. KG, Berlin, Germany) (at BfR); or 400 W Branson Sonifier S-450 CE Digital, (Branson Ultrasonics, St. Louis, Missouri, USA) (IMPB). The sample was cooled in an ice-water bath during sonication [27]. 

### 3.2. ICP-MS Analysis

Measurements were performed with a quadrupole ICP mass spectrometer (iCAP Q, Thermo Fisher Scientific GmbH, Dreieich, Germany) equipped with a PrepFast system (ESI Elemental Service & Instruments GmbH, Mainz, Germany), PFA ST Nebulizer, a quartz cyclonic spray chamber, and a 2.5 mm quartz injector (all from Thermo Fisher Scientific, Waltham, MA, USA) using the following isotopes: ^27^Al and, as an internal standard, ^103^Rh. Calibrations were performed using ionic standards of Al in a 3.5% HNO_3_ solution ranging from 2 to 500 μg/L. The internal standard was added using the ICP-MS PrepFast system. The gas flows for the cooling gas and the auxiliary gas were set to 14 L/min and 0.65 L/min, respectively. The sample flow rate was 0.4 mL/min. All isotopes were analyzed using the collision cell technique at 5 mL/min collision gas flow (93% He and 7% H_2_). Analysis of NM uptake was studied with five replicates per dose. Results were presented as mean values ± standard error of the mean (SEM).

### 3.3. ToF-SIMS Analysis

A dedicated cryogenic sample preparation technique with a high cooling rate was used for sample analysis [28,29]. Liquid propane was cooled using liquid nitrogen, thus preventing evaporation of propane at the contact surface of the immersed specimen. Specimens were in contact with liquid propane for 10 s and were afterwards kept in a frozen state using dry ice. The condenser of the Christ Beta 2-8 lyophilizer (Martin Christ GmbH, Osterode am Harz, Germany) was cooled to −80 °C. The frozen samples were placed on the frozen heating plate, which was inserted in the freeze-drying chamber and heated to −20 °C. Afterwards, vacuum was applied to achieve a pressure of 1.65 mbar. The main drying process started, in which the water is sublimated within 2 h by vacuum and heated to 23 °C. The temperature of 23 °C is maintained for 30 min. The instrument was ventilated and the freeze-dried samples were stored at −80 °C prior to the ToF-SIMS analysis. A ToF-SIMS instrument (ION-TOF V; Ion-TOF GmbH, Münster, Germany) was used for mass spectrometry analyses with a pulsed 30 keV Bi^3+^ liquid metal ion gun (LMIG, direct current (dc), 16 nA). Measurement of cell samples was performed at room temperature. Each spectrum was acquired by scanning the ion beam over a sample area of 400 × 400 µm. Positive secondary ions were collected in the mass range up to *m*/*z* 1200 using 10^6^ Bi^3+^ pulses. Instrument and analysis conditions were used as described elsewhere for the ToF-SIMS analysis of cell membrane lipids [30].

All depth profiles were performed in dual beam mode on a TOF.SIMS V instrument (ION-TOF GmbH, Münster, Germany) of the reflectron-type, equipped with a 30 keV Bi_3_^+^ LMIG as primary ion source, a 20 keV argon gas cluster ion source both mounted at 45° with respect to the sample surface and an electron flood gun. Bi^3+^ was selected as primary ion by appropriate mass filter settings. Primary and sputter ion currents were directly determined at 200 μs cycle time (i.e., a repetition rate of 5.0 kHz) using a Faraday cup located on a grounded sample holder. Scanning area for analysis was 200 × 200 μm^2^ with 512 × 512 pixels. The sputter area for each measurement was 1000 μm × 1000 µm. Surface charging was compensated by flooding with low energy electrons. 

ToF-SIMS depth profiles were acquired in positive ion mode. The mass scale was internally calibrated using a number of well-defined and easily assignable secondary ions (C_2_H_5_^+^, C_3_H_7_^+^, and C_4_H_9_^+^) keeping the error of calibration for all spectra below 5 ppm. The data were evaluated using the Surface Lab software (ION-TOF GmbH, Münster, Germany). 

Statistical analyses of the ToF-SIMS data were performed as described in detail elsewhere [30,31,32,33,34]. In brief, the acquired data were binned to 1 u. Data processing was carried out with the statistical package SPSS + (version 21) using the mass range between 200 and 1200 mass units to detect significant differences between treated and untreated cells. Ions lower than mass 200 were excluded from the study to avoid contamination of the ions from salts, system contaminants, and other medium components. Each acquired spectrum was then normalized, setting the peak sum to 100%. A principal component analysis (PCA) was performed using all ions. To show that data sets could be separated with a supervised model from each other a Fisher’s discriminant analysis was performed. The performance of the discriminant model was verified by applying the cross-validation procedure based on the “leave-one-out” cross-validation formalism.

## 4. Conclusions

The ToF-SIMS measurements have shown that aggregation and incorporation of Al NMs in HaCaT cells are influenced by treatment with vitamins. Experiments with retinol led to the formation of large Al aggregates, which are intercalated in the membrane regions of the cells, while vitamin D3 treatments resulted in the formation of small agglomerates within the entire cell. These findings suggest that, depending on the vitamin treatment, different pathways are used for the uptake of Al NMs. 

Furthermore, the results show a decreased uptake rate of Al_2_O_3_ NMs after treatment with vitamins (retinol and vitamin D3) in comparison with the control as well as with the exposure to Al^0^ NMs in combination with both vitamins. A likely explanation for this behavior is the change in the lipid membrane composition, which might lead to an enhanced rigidity of the membrane. Treatment with either retinol or vitamin D3 leads to a drastic decrease in the uptake of Al_2_O_3_ NMs.

In contrast to the protective effect observed for Al_2_O_3_ NMs, ToF-SIMS measurements revealed a changed lipid metabolite profile for cells in response to co-exposure to vitamin D3 and Al^0^ NMs. The metabolic changes led to a shift in the PC-to-PE ratio, which contributes to an increased uptake of Al NMs due to a leaky cell membrane. 

We conclude a protective effect of the vitamins A and D3 for cells, which are in contact with nanoparticulate oxides such as Al_2_O_3_. On the other hand, the presence of these substances may slightly promote the uptake of metallic NMs. Our findings also reflect the high importance of a thorough physicochemical particle characterization, as parameters like agglomeration, solubility, and biokinetics may affect the uptake of NMs with the same elemental constituent.

## Figures and Tables

**Table 1:** Characterization data for Al^0^ and Al_2_O_3_ nanomaterials (NM). Modified from [12].

Methods	Al^0^ NM	Al_2_O_3_ NM
TEM	Primary particle size and shape: 2–50 nm, nearly spherical	Primary particle size and shape: 10 × 20–50 nm, grain-like shape
EELS-TEM	Core-shell structure, thin (2–5 nm) oxide layer	Fully oxidized particle
XRD	Aluminum surface; partially oxidized	Fully oxidized surface
SAXS	Particle size: >20 nm	Primary particle size: 14.2 nm Aggregates’ size: >20 nm
SP-ICP-MS	Primary particle size: 54–80 nm	Primary particle size: 50–80 nm
ICP-MS	Ion release: 0.2–0.5% (in 0.05% BSA)	Ion release: 0.2–0.4% (in 0.05% BSA)

**Table 2:** Significantly increased cell membrane constituents upon treatment of HaCaT cells with Al^0^ NMs divided by the additional treatment of either retinol low or high vitamin D3.

Retinol	Low Vitamin D3	High Vitamin D3
diacylglycerols	phosphatidylethanolamines	lyso-phosphatidylcholines
lyso-phosphatidic acids	-/-	-/-

**Table 3:** Significantly increased cell membrane constituents upon treatment of HaCaT cells with Al_2_O_3_ NMs divided by the additional treatment of either retinol low or high vitamin D3.

Retinol	Low Vitamin D3	High Vitamin D3
diacylglycerols	diacylglycerols	Diacylglycerols
lyso-phosphatidic acids	lyso-phosphatidylcholines	phosphatidic acids
-/-	dihydroceramides	diacylglycerol phosphates

## Supplementary Materials

The following are available online at https://www.mdpi.com/1422-0067/21/4/1278/s1.

## Abbreviations

Al	aluminum
DAG DLS EELS	diacylglycerol dynamic light scattering electron energy loss spectroscopy
EFSA	European Food Safety Authority
HaCaT	human keratinocyte cell line
ICP-MS	inductively coupled plasma mass spectrometry
NMs	nanomaterials
PA	phosphatidic acids
PC	phosphatidylcholine
PCA	principal component analysis
PE	phosphatidylethanolamine
PIP2	phosphatidylinositol 4,5-bisphosphate
PKC SAXS SEM SP TEM	protein kinase C small angle X-ray scattering standard error of the mean single particle transmission electron microscopy
ToF-SIMS	time-of-flight secondary ion mass spectrometry
TWI XRD	tolerable weekly intake X-ray diffraction
