# Supplementary material for: The Vitamin A and D Exposure of Cells Affects the Intracellular Uptake of Aluminum Nanomaterials and Its Agglomeration Behavior: A Chemo-Analytic Investigation
Source: Int J Mol Sci. 2020 Feb 14;21(4):1278. doi: 10.3390/ijms21041278 (PMC7072912; doi:10.3390/ijms21041278)
Supplement: Supplementary file 1 [file ijms-21-01278-s001.pdf]

All graphs in the Supplementary Material sections display the metabolic changes of HaCaT cell exposed to Al^0 NM, Al_2O_3 NM and $AlCl_3*6H_2O$ as well as retinol and/or low or high vitamin D3. The results showed for the compounds, which loaded high on factor 1 ($x \geq 0.95$) and therefore are mainly responsible for group separation. For the relative intensity, the mean of the control + retinol group for unexposed HaCaT cells was taken as 100% in all cases. ***: $p \leq 0.05$.

5.1 Non-additive, particle specific metabolic effects in co-exposure experiments with Al_2O_3 NM, Al^0NM, $AlCl_3*6H_2O$ and retinol

Synergistic effects could be observed in co-exposition experiments of Al^0 NM and Al_2O_3 NM and retinol only, but not in co-exposition experiments with $AlCl_3*6H_2O$ and retinol (see Figure 9).

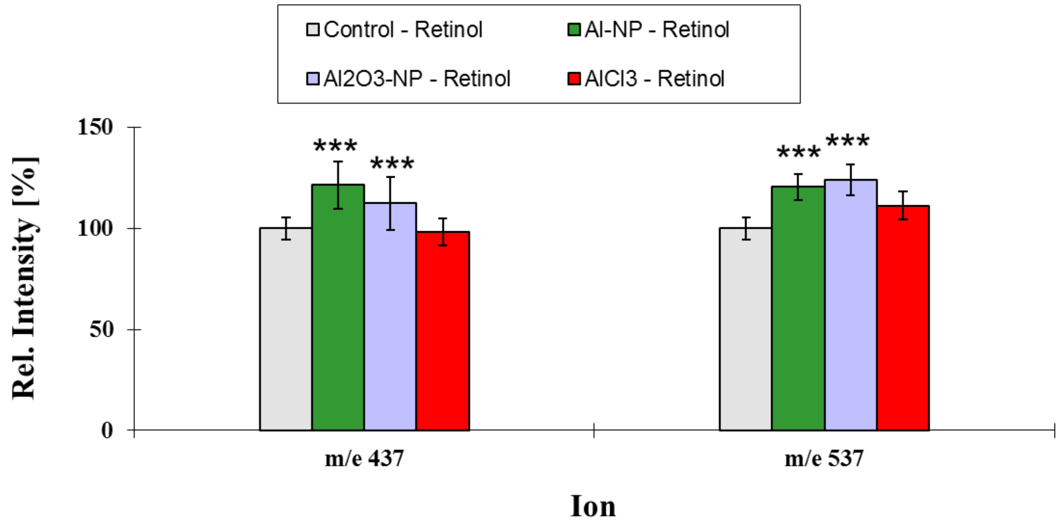

Figure S1: Histogram of ion yields for m/e 437 (lyso-phosphatidic acid, Lyso-PA (C18:1)) and m/e 537 (diacylglycerol DG (C30:2))

Additionally synergistic effects can be seen in co-exposure experiments with Al_2O_3 NM and retinol where co-exposition experiments with $AlCl_3*6H_2O$ and retinol show antagonistic effects and co-exposition experiments with Al^0 NM and retinol show similar metabolite level to HaCaT cell, exposed with retinol only (see Figure 10).

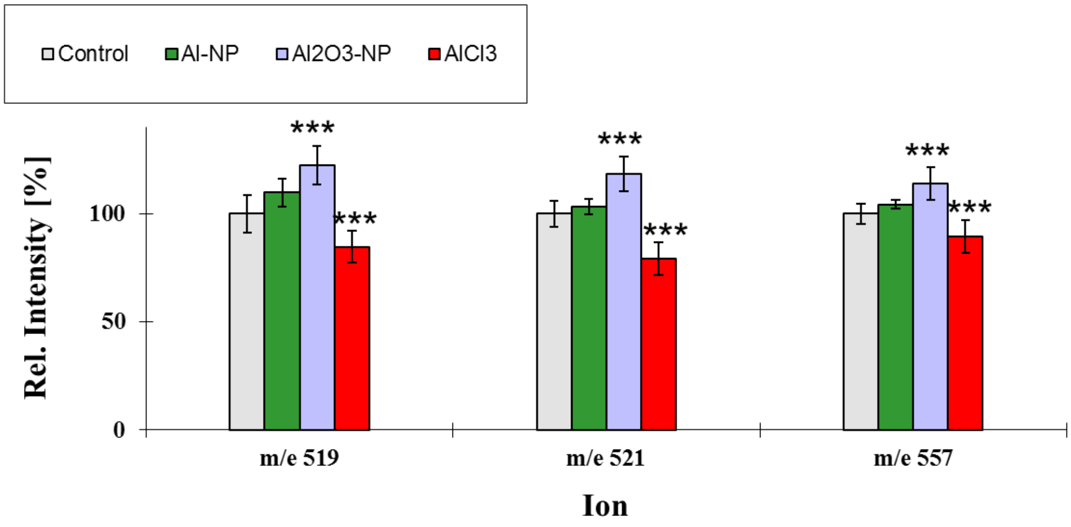

Figure S2: Histogram comparisons of ion yields for ion m/e 519 (lyso-phosphatidic acid, Lyso-PA (C24:2)), ion m/e 521 (lyso-phosphatidic acid, Lyso-PA (C24:1)) and ion m/e 521 (diacylglycerol DG (C32:6)).

*5.2 Non-additive, particle specific metabolic effects in co-exposure experiments with Al_2O_3 NM, Al^0NM, $AlCl_3$*$6H_2O$ and low vitamin D3*

Specific synergistic effects with ionic $AlCl_3$*$6H_2O$ and Al_2O_3 NM in combination with low vitamin D3, which could not be observed in co-exposure experiments of Al^0 NM and low vitamin D3 (see Figure 11) were detected.

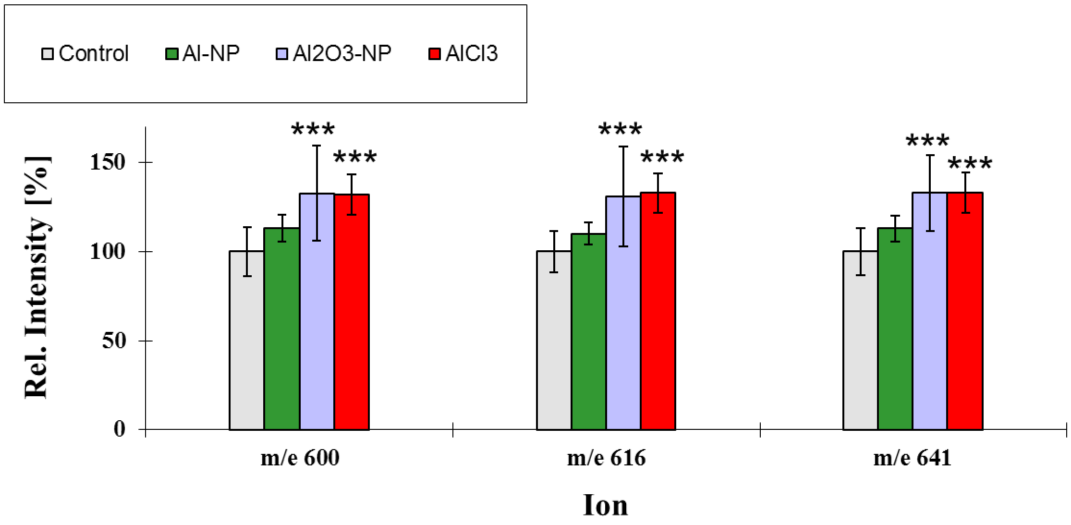

Figure S3: Histogram comparisons of ion yields for ion m/e 600 (lyso-phosphatidylcholine, Lyso-PC (C24:4)), ion m/e 616 (diacylglycerol DG (C36:5)) and ion m/e 641 (dihydroceramide Cer (tC40:0)).

Additionally a synergistic effect could only be observed in co-exposure experiments with Al^0 NM and low vitamin D3 (see Figure 12).

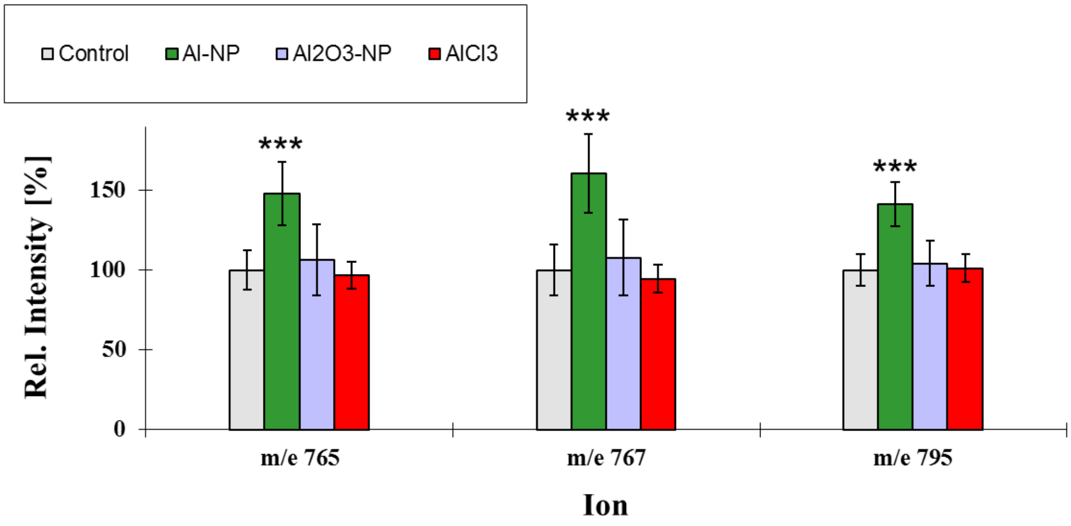

Figure S4: Histogram comparisons of ion yields for Ion m/e 765 (phosphatidylethanolamine, PE (C38:6)), ion m/e 767 (phosphatidylethanolamine, PE (C38:5)) and ion m/e 795 (phosphatidylethanolamine, PE (C40:5)).

*5.3 Non-additive, particle specific metabolic effects in co-exposure experiments with Al_2O_3 NM, Al^0NM, $AlCl_3$*$6H_2O$ and high vitamin D3.*

Synergistic effects with Al_2O_3 NM in combination with high vitamin D3, which could not be observed in co-exposure experiments of all other combinations, were found (see Figure 13).

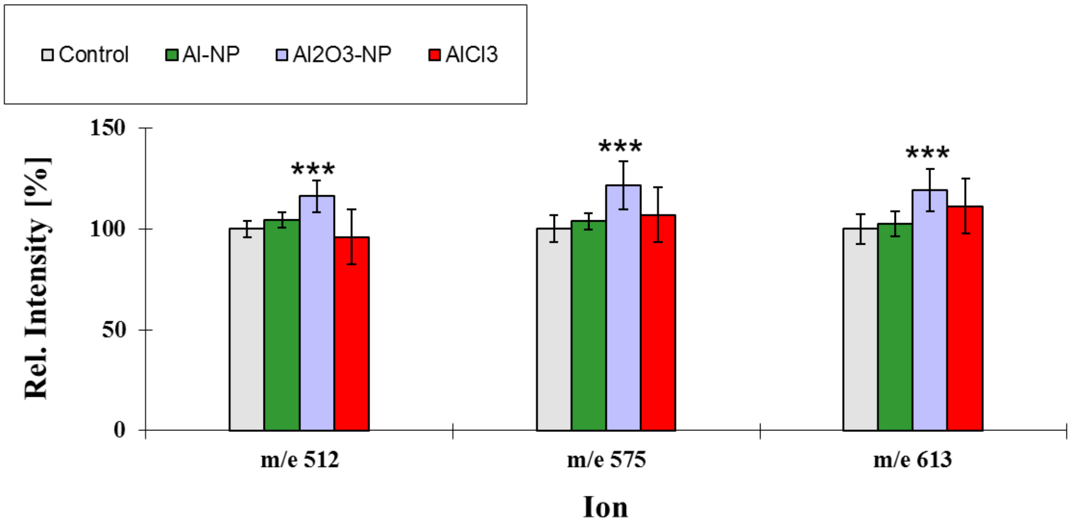

Figure S5: Histogram comparisons of ion yields for ion m/e 512 (lyso-phosphatidylserine, Lyso-PS (O-C18:0)), ion m/e 575 (diacylglycerol phosphate, DG (P-C34:3)) and ion m/e 613 (diacylglycerol, DG (C36:6)).

Additionally a synergistic and antagonistic effect could only be observed in co-exposure experiments with Al^0 NM and high vitamin D3 (see Figure 14).

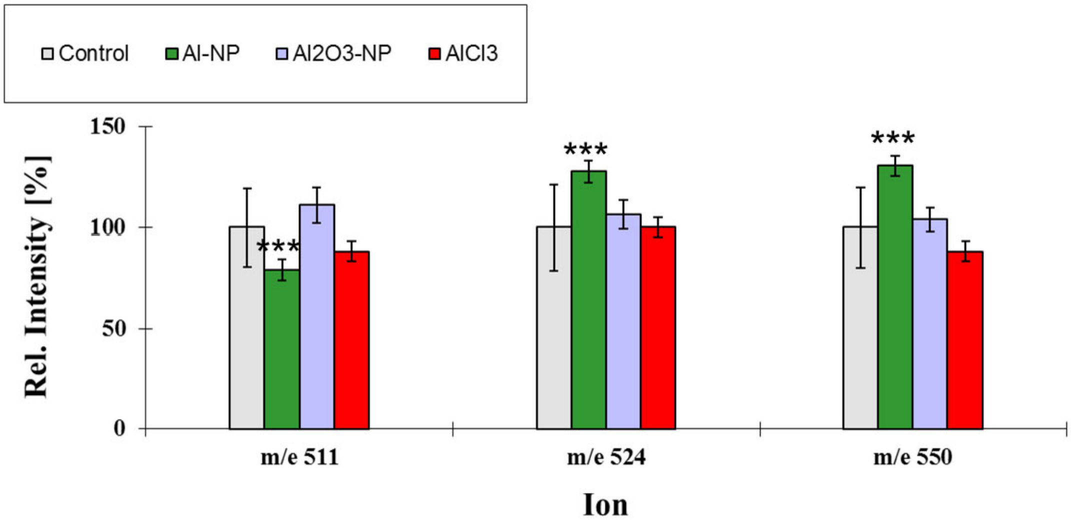

Figure S6: Histogram comparisons of ion yields for ion m/e 511 (diacylglycerol, DG (C28:1)), ion m/e 524 (lyso-phosphatidylcholine, Lyso-PC (C18:0)) and ion m/e 550 (lyso-phosphatidylcholine, Lyso-PC (C20:1)).
